# Phenotypic Screening and Organ-Specific Transcriptomics Unveil Diverse Salt Tolerance Responses at the Seedling Stage in Wheat (*Triticum aestivum* L.)

**DOI:** 10.3390/plants15121905

**Published:** 2026-06-19

**Authors:** Wenjia Zhang, Jinpeng Zou, Yinying Wu, Ningjun Hu, Shengyuan Lv, Xiukun Liu, Xiaoyan Duan, Danping Li, Haosheng Li, Jianjun Liu, Xinyou Cao, Wujun Ma, Xueyan Chen, Xin Gao

**Affiliations:** 1 Shandong Academy of Agricultural Sciences, Jinan 250100, China; zhangwenjia@saas.ac.cn (W.Z.); zoujinpeng@saas.ac.cn (J.Z.);; 2 Crop Research Institute, Shandong Academy of Agricultural Sciences/National Engineering Research Center of Wheat and Maize/State Key Laboratory of Wheat Improvement/Key Laboratory of Wheat Biology and Genetic Improvement in North Yellow & Huai River Valley/Shandong Provincial Technology Innovation Center for Wheat, Jinan 250100, China; 3 College of Agronomy, Qingdao Agricultural University, Qingdao 266109, China; 4 Institute of Crop Germplasm Resources, Shandong Academy of Agricultural Sciences, Jinan 250100, China

**Keywords:** wheat (*Triticum aestivum* L.), salt tolerance, organ-specific divergence, comparative transcriptomics, DEGs

## Abstract

Identifying superior salt-tolerant germplasm and resistance genes is crucial, as wheat (*Triticum aestivum* L.) seedlings are highly vulnerable to salt stress. Here, using an optimized 150 mM NaCl treatment, we screened 137 Chinese wheat accessions via an organ-specific method. Phenotyping analysis revealed extensive organ-specific divergence, with 48.91% of accessions displaying inconsistent performance between shoot and root length. We then performed comparative transcriptomics on three representative phenotypes at the seedling stage: Gaoyou 2018, representing the salt dual-sensitive group; Huapei 5, representing the salt dual-tolerant group; and Jimai 60, representing the divergent group with higher tolerance in shoots rather than in roots. Analysis of overlapping differentially expressed genes (DEGs) across all three accessions revealed a basal stress response—characterized by induced osmotic defense and suppressed primary growth—exemplifying a classical growth–defense trade-off. Genotype-specific DEG profiling demonstrated that the divergent Jimai 60 maintains its shoot advantage by reinforcing physical barriers and inhibiting apoptosis. Conversely, transcriptomic profiling implies that the systemically tolerant Huapei 5 maintains coordinated shoot and root tolerance at the seedling stage by strongly activating below-ground Na^+^ homeostasis (efflux and compartmentalization) while simultaneously down-regulating non-essential immune responses to optimize defense energy reallocation. Collectively, our findings provide novel insights into the organ-differentiated salt tolerance of wheat, offering well-characterized elite germplasm and compelling genetic targets for future molecular breeding.

## 1. Introduction

Wheat (*Triticum aestivum* L.) is one of the most important staple crops globally, playing a critical role in ensuring food security [1]. However, soil salinization has emerged as a major abiotic stress, severely constraining crop yield and sustainable agriculture [2,3]. In China, saline–alkali lands cover approximately 36.7 million hectares, and the proportion of arable land affected by salinity continues to expand annually due to climate change and other factors [4,5]. High-salinity environments cause osmotic stress, ion toxicity, and oxidative damage in plants [6,7]. As a typical salt-sensitive crop, wheat is susceptible to salt stress throughout its life cycle, with the early seedling stage being particularly sensitive [8]. Therefore, extensively evaluating the natural phenotypic variation during the seedling stage, identifying elite salt-tolerant germplasm, and uncovering the underlying molecular networks are of great practical significance for accelerating the breeding of new salt-tolerant wheat varieties.

Plant responses to salt stress involve a complex spatiotemporal process regulated by multiple genes. The initial osmotic stress primarily inhibits root elongation and new leaf expansion, while subsequent ionic toxicity leads to the excessive accumulation of toxic ions in older leaves, triggering chlorosis and necrosis [9,10]. Given that roots and shoots often exhibit significant differences in perceiving and responding to salt stress, an individual phenotypic trait cannot fully reflect genuine salt tolerance. Thus, comprehensive evaluations based on organ-specific growth traits are essential. Moreover, prior to large-scale population phenotyping, establishing an optimal salt stress concentration is crucial for capturing actual genetic variation. Because wheat accessions differ vastly in their tolerance thresholds, excessively high concentrations cause universal lethality, while low concentrations fail to induce significant phenotypic variations [11,12]. Therefore, determining the appropriate stress concentration through preliminary gradient assays and subsequently screening a large-scale, genetically diverse natural population is an effective method to accurately identify extreme salt-tolerant and sensitive accessions [13,14]. This systemic screening approach not only reveals the extent of salt tolerance variation in existing germplasm but also provides a contrasting genetic foundation for downstream mechanistic studies.

Once extreme phenotypes are identified, transcriptome sequencing serves as a powerful tool to mine core stress-responsive genes and adaptive pathways [15,16]. Previous studies have made substantial progress, revealing that up-regulated genes under salt stress are mainly enriched in signal transduction, phytohormone pathways (e.g., abscisic acid, ABA), osmotic adjustment, ion transport, and reactive oxygen species (ROS) homeostasis [17,18,19]. However, systematic transcriptomic studies investigating the balance between basic survival and plant growth (growth–defense trade-off) in wheat seedlings remain limited. Compared to analyzing a single accession with a complex genetic background, comparative transcriptomic profiling of both shoots and roots at the seedling stage across extreme genotypes with different organ-specific traits (e.g., salt-tolerant vs. organ-divergent) offers distinct advantages. This method effectively filters out background noise and helps precisely distinguish the basal defense response of wheat from the specific regulatory networks that determine salt tolerance variation.

In summary, although progress has been made in salt tolerance phenotyping and functional genomics in wheat, studies integrating large-scale, multi-organ population screening with deep transcriptomics of extreme materials at an early stage are still required. In this study, we first established 150 mM NaCl as the optimal stress concentration for the wheat seedling stage. Subsequently, we systematically evaluated the salt tolerance of a natural population containing 137 wheat accessions and successfully classified them into four distinct salt-response groups based on phenotypic variations between shoots and roots. Based on these results, three representative cultivars from different groups exhibiting distinct phenotypes at the seedling stage were selected: Gaoyou 2018 (salt dual-sensitive group), Huapei 5 (salt dual-tolerant group), and Jimai 60 (divergent type: exhibiting higher tolerance in shoots than in roots). Transcriptome sequencing was then performed to analyze their gene expression profiles in both shoots and roots under salt stress. This study aims to identify core pathways and key candidate genes and to provide new insights into the molecular mechanisms of wheat salt tolerance at an early stage, offering a theoretical reference for screening and utilizing elite germplasm resources.

## 2. Results

### 2.1. Screening of Appropriate Salt Concentration for Wheat Seedling Salt Tolerance Evaluation

**Figure 1:**
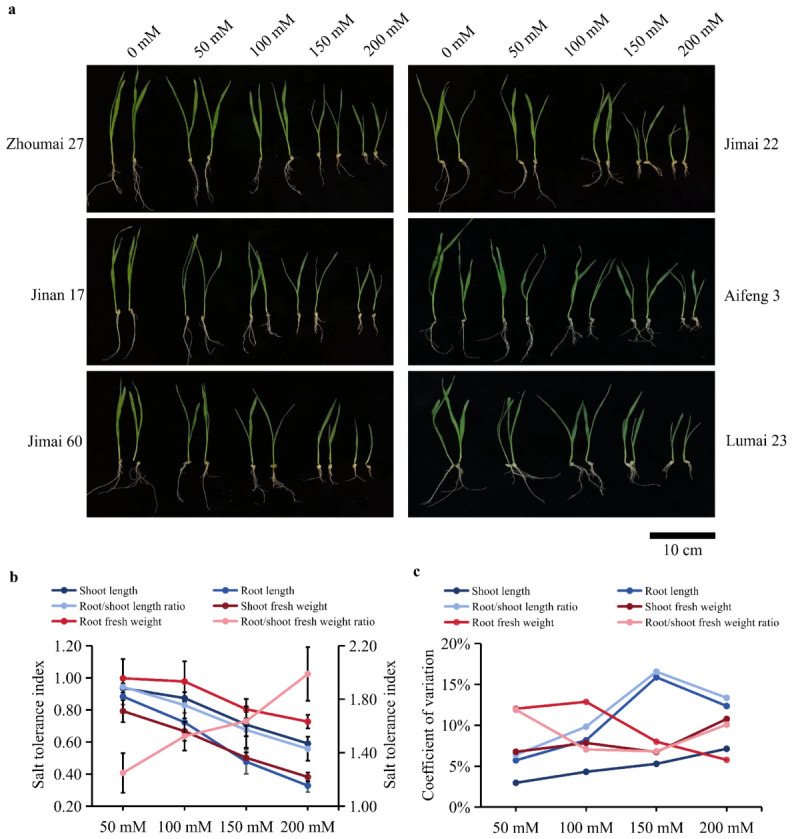
Growth performance and salt tolerance index (STI) of six wheat cultivars under different salt concentrations. (**a**) Seedling growth under different salt concentrations (0, 50, 100, 150 and 200 mM, from left to right); (**b**) STI of shoots and roots under different salt concentrations. STI was calculated as the value under a salt concentration divided by the value under the control (0 mM). The X-axis indicates salt concentrations (50, 100, 150, 200 mM). The left primary Y-axis represents the STI of shoot length, root length, shoot fresh weight, root fresh weight, and root/shoot length ratio, while the right secondary Y-axis represents the STI of root/shoot fresh weight ratio. Data are presented as the mean ± SD of six wheat cultivars at each salt concentration (*n* = 6); (**c**) coefficient of variation (CV) of STI for shoots and roots under different salt concentrations. The X-axis indicates salt concentrations (50, 100, 150, 200 mM). The Y-axis represents the CV of each trait, calculated as SD/mean. Five seedlings were measured per replicate. Bar = 10 cm.

Determining an appropriate stress concentration is crucial for evaluating genetic variation in salt tolerance within a plant population. Excessively high concentrations can cause widespread mortality, whereas low concentrations may fail to induce distinguishable phenotypic differentiation. We selected six representative wheat cultivars (Zhoumai 27, Jinan 17, Jimai 60, Jimai 22, Aifeng 3, and Lumai 23) with extensive phenotypic variation. A preliminary seedling experiment was conducted using four NaCl stress concentrations (50, 100, 150, and 200 mM) and a 0 mM control group (Table S1). The results demonstrated a dose-dependent inhibition of seedling growth. With increasing stress concentrations, the values of measured phenotypic traits continuously decreased (Figure 1a). Under the 150 mM NaCl treatment, plants maintained basal growth despite the stress, whereas under 200 mM NaCl treatment, seedlings exhibited severe growth retardation and wilting, suggesting this level approaches the lethal threshold for certain accessions (Figure 1a). We further evaluated the degree of stress injury at the seedling stage under different salt concentrations based on the salt tolerance index (STI). With increasing stress concentrations, the STI of all measured traits exhibited a continuous decline, with the exception of the root/shoot fresh weight ratio, indicating that shoot fresh weight is relatively more sensitive to severe salt stress (Figure 1b).

To evaluate phenotypic variation among cultivars at different concentrations, we calculated the CVs for the STI of different traits (Table S2). Under mild salt stress (50 and 100 mM NaCl), the CVs were small, masking potential tolerance differences among cultivars. Under moderate salt stress (150 mM), the CVs for root length and root/shoot length ratio reached their maximum (Figure 1c; Table S2), indicating the greatest phenotypic differentiation and highest screening efficiency for salt-tolerant accessions. Meanwhile, the CVs of shoot fresh weight, root fresh weight, and other traits remained at a moderate level (Figure 1c; Table S2). Based on the comprehensive analysis of phenotypic traits and variations, the 150 mM NaCl treatment effectively revealed genetic-dependent differences in salt tolerance. Thus, it was selected as the optimal concentration for subsequent large-scale population screening in this study.

### 2.2. Phenotypic Variation in Shoot and Root Length in 137 Wheat Accessions

Based on the preliminary assay establishing the 150 mM NaCl treatment as the test conditions, we performed a large-scale salt tolerance screening and phenotypic variation analysis on a natural population comprising 137 Chinese wheat accessions. Shoot length and root length were chosen as key evaluation traits, since growth inhibition represents the most sensitive and earliest response to salt stress [8]. Under normal growth conditions (control group; 0 mM NaCl), the population exhibited basal genetic diversity. For the 137 accessions, group means are presented as mean ± SD: shoot lengths ranged from 11.88 to 23.40 cm (CV = 13.56%), and root lengths ranged from 7.76 to 19.58 cm (CV = 18.61%) (Table S3). Under the 150 mM NaCl treatment, the absolute phenotypic values narrowed due to overall growth inhibition (9.00–15.20 cm for the range of shoot length and 4.00–9.14 cm for the range of root length), and the CVs experienced an expected slight decrease (10.85% and 15.35% for shoot and root, respectively) (Table S3). Nevertheless, the CVs of the stressed population remained at a substantial level (>10%), indicating that the 150 mM treatment effectively induced measurable tolerance differentiation within the population.

Furthermore, comparisons revealed that the phenotypic CV of root length was consistently greater than that of shoot length under both control and stress conditions. This implies that roots, as the primary organ in direct contact with saline substrates, exhibit more diverse physiological responses among different genotypes. Overall, the 137 accessions displayed a large amplitude of genetic variation, confirming their suitability for identifying extreme salt-tolerant and sensitive germplasm.

### 2.3. Population Screening and Identification of Extreme Variant Accessions

**Figure 2:**
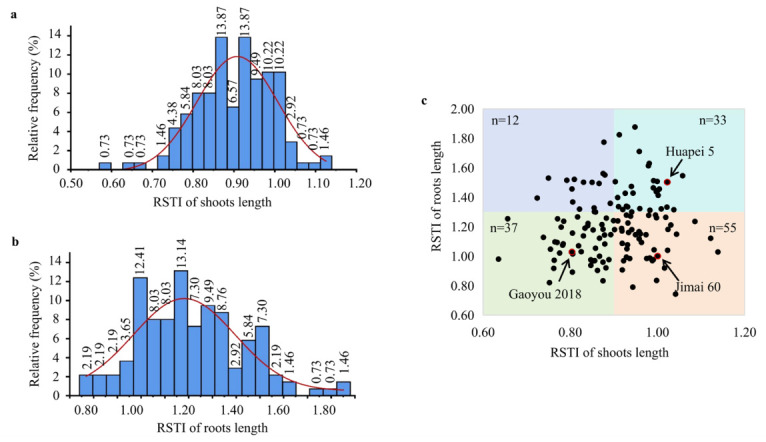
Statistical analysis of relative salt tolerance index (RSTI) in 137 wheat accessions. (**a**,**b**) Frequency distribution histogram of RSTI for shoot length (**a**) and root length (**b**). The Y-axis represents the relative frequency of the RSTI trait across different group spacing in the 137 wheat accessions, and the X-axis represents the RSTI of shoot length (**a**) and RSTI of root length (**b**). The red line represents the fitted density curve. RSTI was normalized to Jimai 60; (**c**) scatter plot of RSTI between shoot and root length. Each black dot represents a wheat accession. Background colors indicate the quadrant classification: light blue, dual-tolerant; light red, higher tolerance in shoots; light purple, higher tolerance in roots; light green, dual-sensitive. Five seedlings were measured per replicate.

Identifying extreme genotypes under an optimal stress concentration lays the foundation for downstream transcriptomic investigations. We first calculated the relative salt tolerance index (RSTI) based on shoot length and root length (Figure 2a,b). Frequency distribution analysis indicated continuous variation conforming to polygenic quantitative traits. The broader distribution of the root length RSTI corroborated the observation of higher root length variability.

To characterize resistance divergence at the organ level, we mapped a two-dimensional evaluation matrix based on shoot length and root length RSTI. Accordingly, the salt tolerance responses at the seedling stage of the natural population were classified into four groups: (I) salt-sensitive (27.01%, 37/137); (II) divergent phenotype: exhibiting higher tolerance in shoots (40.15%, 55/137); (III) divergent phenotype: exhibiting higher tolerance in roots (8.76%, 12/137); and (IV) salt-tolerant (24.09%, 33/137) (Figure 2c). Relying on this matrix, we selected three representative extreme cultivars possessing high phenotypic contrast and diverse geographical origins for subsequent transcriptomic analysis: Gaoyou 2018 (both shoot- and root-sensitive, hereafter referred to as dual-sensitive type) originated from Hebei Province, Huapei 5 (both shoot- and root-tolerant, hereafter referred to as dual-tolerant type) originated from Henan Province, and Jimai 60 (divergent type, with higher tolerance in shoots than in roots) originated from Shandong Province.

### 2.4. Transcriptome Sequencing Evaluation and Global Expression Patterns in Response to Salt Stress

To elucidate the transcriptional basis of how these distinct extreme cultivars respond to salinity, we sequenced the shoots and roots of Gaoyou 2018, Huapei 5, and Jimai 60 under normal control and 150 mM NaCl conditions (totaling 36 samples). The transcriptome sequencing was performed with paired-end reads, with a mean read length of 150 bp. Libraries were constructed using the poly(A) selection method. Sequencing output showed that each library yielded 69.99 to 98.97 million high-quality effective reads with average GC contents between 50% and 60% (Table S5). The unique mapping rates to the wheat reference genome exceeded 80% for all samples, and the Q30 values were consistently > 95%, demonstrating robust sequencing data quality (Table S5). Principal component analysis (PCA) displayed tight clustering among the three biological replicates under matching conditions. The control groups distinctly separated from the salt-stressed groups; moreover, shoot samples clustered separately from root samples across principal axes, underscoring evident organ-specific transcriptional disparities (Figure S1a).

DEG analysis highlighted extensive stress-induced transcriptomic shifts. For the dual-sensitive material Gaoyou 2018, 1147 DEGs (470 up-regulated, 677 down-regulated) were identified in shoots, and 8284 DEGs (4111 up-regulated, 4173 down-regulated) in roots (Figure S1b; Tables S6–S8). By contrast, the dual-tolerant Huapei 5 mounted a broader response, identifying 6318 DEGs in shoots (3204 up-regulated, 3114 down-regulated) and 12,656 in roots (5680 up-regulated, 6976 down-regulated) (Figure S1b; Tables S6, S9 and S10). The divergent Jimai 60 displayed intermediate levels: 3894 DEGs in shoots (2292 up-regulated, 1602 down-regulated) and 9316 in roots (4076 up-regulated, 5240 down-regulated) (Figure S1b; Tables S6, S11 and S12). When facing an equivalent 150 mM NaCl stress, we observed a trend that the number of DEGs roughly aligned with the phenotypic salt tolerance (Gaoyou 2018 < Jimai 60 < Huapei 5). This pattern implies that more tolerant genotypes may tend to initiate more active transcriptional reprogramming. Consistently across cultivars, DEGs identified in roots substantially outnumbered those in shoots.

### 2.5. DEG Classification Based on Extreme Phenotypes

**Figure 3:**
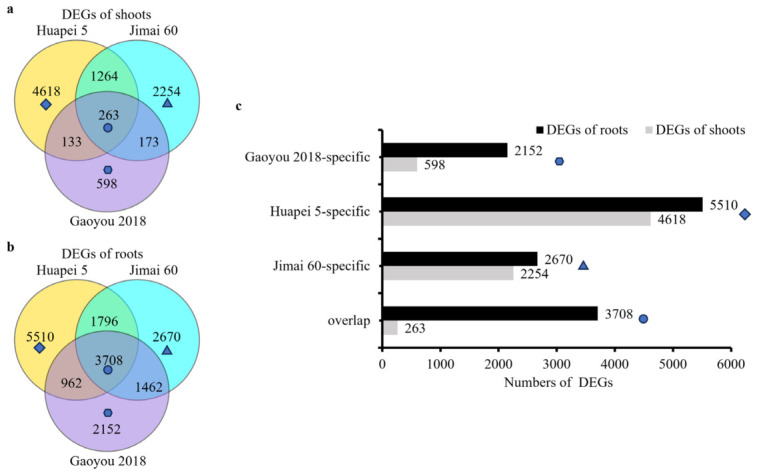
Statistics of differentially expressed genes (DEGs) in three representative wheat cultivars under 150 mM salt treatment. (**a**,**b**) Venn diagrams showing the overlap of DEGs in shoots (**a**) and roots (**b**) of Huapei 5, Jimai 60 and Gaoyou 2018 under 150 mM salt treatment compared with the control (0 mM). (**c**) Bar graph showing the numbers of common DEGs and cultivar-specific DEGs in shoots and roots of the three cultivars. Among them, black bars and gray bars represent DEGs in roots and shoots, respectively. Note: circle, triangle, rhombus and hexagon represent the common DEGs of the three varieties and the specific DEGs of Jimai 60, Huapei 5 and Gaoyou 2018, respectively.

Given the diverse salt tolerance phenotypes exhibited by the three selected wheat cultivars, we hypothesized that the DEGs across varying genotypes and tissues carry distinct biological implications. Consequently, we performed an intersection analysis using Venn diagrams on the DEGs from both the shoots and roots of Gaoyou 2018, Huapei 5, and Jimai 60. The results demonstrated significant genotype-specific distributions and intersections of DEGs. Notably, the total number of DEGs in roots consistently exceeded that in shoots across all materials, implying a more extensive transcriptional response to salinity in the root system (Figure 3a,b).

To effectively identify crucial tolerance genes, we classified the gene pools into three representative target groups: (1) Overlapping DEGs (genes commonly responsive across all the three cultivars; 263 in shoots and 3708 in roots) (Figure 3a,b; Table S13). This group is largely independent of specific genetic backgrounds and may represent the basal transcriptional response of wheat seedlings to salt stress. (2) Jimai 60-specific DEGs (genes exclusively differentially expressed in the divergent accession Jimai 60; 2254 in shoots and 2670 in roots) (Figure 3; Table S14). Analyzing this group can elucidate the potential regulatory mechanisms governing its shoot tolerance. (3) Huapei 5-specific DEGs (genes exclusively differentially expressed in the dual-tolerant accession Huapei 5; 4618 in shoots and 5510 in roots) (Figure 3; Table S14). These genes are likely integral to the tolerance phenotype and serve as key candidates for the discovery of elite resistance genes. This multi-dimensional classification reduces background interference from diverse genetic backgrounds, laying a solid groundwork for targeted candidate gene selection. Additionally, we also analyzed Gaoyou 2018-specific DEGs (genes exclusively differentially expressed in the dual-sensitive accession Gaoyou 2018; 598 in shoots and 2152 in roots) (Table S14), aiming to clarify the specific transcriptional response characteristics of this dual-sensitive accession to salt stress.

### 2.6. The Basal Defensive Line: Overlapping Stress-Responsive Network

**Figure 4:**
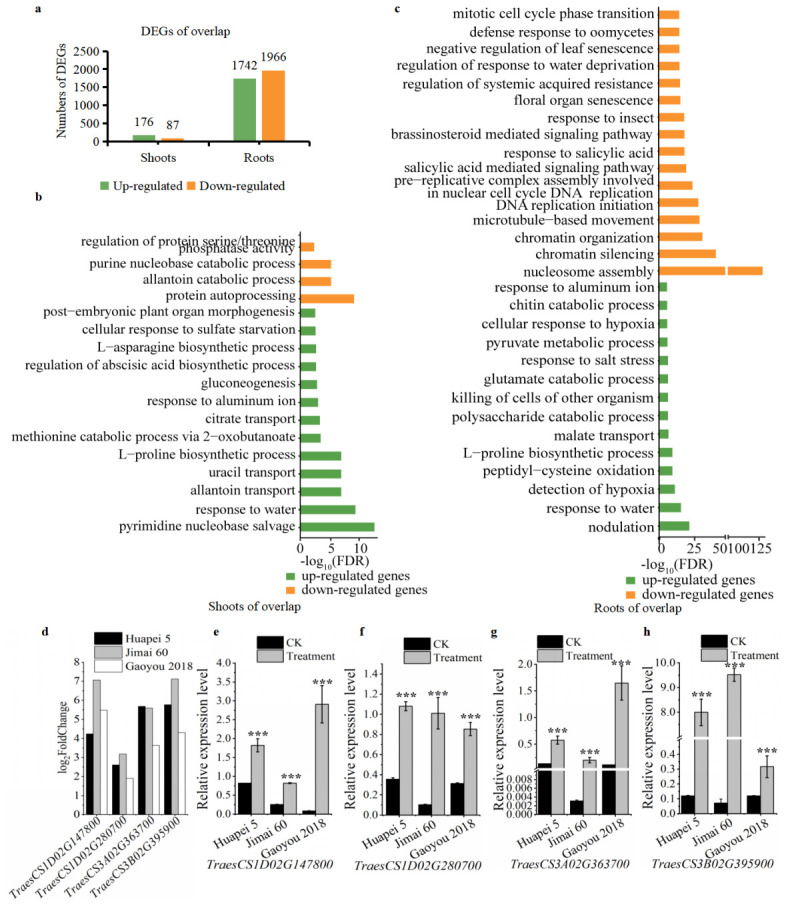
Overlap of differentially expressed genes (DEGs) among three representative wheat cultivars under 150 mM salt treatment. (**a**) Numbers of common DEGs in Huapei 5, Jimai 60, and Gaoyou 2018 in shoots and roots. Green and yellow bars represent up-regulated and down-regulated DEGs, respectively; (**b**,**c**) GO enrichment analysis of common DEGs in shoots (**b**) and roots (**c**) of the three cultivars under 150 mM salt treatment compared with control (0 mM). Green and yellow bars represent up-regulated and down-regulated DEGs, respectively. *p*-values were adjusted by the Benjamini–Hochberg correction, and only statistically significant GO categories (FDR < 0.05) are shown. (**d**–**h**) Transcriptomic log_2_FoldChange values (**d**) and qRT-PCR-based expression patterns in shoots (**e**–**h**) for four key genes involved in L-proline biosynthesis and ABA-responsive pathways under salt stress. Data were obtained from three wheat cultivars (Huapei 5, Jimai 60, Gaoyou 2018); the expression of *ACTIN* was used to normalize mRNA levels. The values are means (±SE) of three biological replicates. *** indicates significant difference at *p* < 0.001 relative to the 0 mM control.

As outlined, overlapping DEGs may denote the basal transcriptional response of wheat to salinity at the seedling stage. In shoots, 263 genes responded uniformly (176 up-regulated, 87 down-regulated), while in roots this shared response engaged 3708 genes (1742 up-regulated, 1966 down-regulated) (Figure 4a; Table S13). This pronounced disparity indicates that root tissues likely involve a more comprehensive regulatory network during basic salt stress adaptation.

To deduce potential functions, GO enrichment analysis revealed disparate characteristics between the organs. (1) Osmotic adjustment and metabolic reallocation in shoots: Up-regulated overlapping DEGs in shoots significantly enriched in the ABA biosynthetic process, L-proline biosynthesis, responses to water deprivation, and organic acid transport (Figure 4b). This implies that under 150 mM salinity, shoots predominantly counteract osmotic stress by triggering proline accumulation and ABA pathways. Meanwhile, commonly down-regulated shoot genes were linked to protein phosphorylation modification and nitrogenous compound catabolism, hinting at a systemic reallocation of basal signaling and metabolic routes (Figure 4b). (2) Cell cycle inhibition and energy metabolism alterations in roots: Down-regulated overlapping DEGs in roots clustered prominently around nuclear cycle progression (e.g., mitotic cell cycle phase transition, DNA replication initiation, and chromatin remodeling) alongside brassinosteroid signaling pathways (Figure 4c). This outcome implies a generalized salt-induced suppression of root cell division and DNA replication, constraining normal growth. Conversely, shared up-regulated root genes localized into energy-consuming catabolic processes (such as pyruvate, polysaccharide, and glutamate catabolism) and chitin degradation. We postulate that root systems may escalate carbohydrate catabolism to provision the additional energy essential for sustaining basal physiological viability during stress.

To validate the reliability of our transcriptomic data, four key shoot-expressed DEGs were selected for qRT-PCR based on the enrichment results showing that up-regulated shared DEGs in shoots were prominently enriched in L-proline biosynthesis and ABA-responsive pathways. These included three genes (*TraesCS3B02G395900*, *TraesCS3A02G363700, TraesCS1D02G280700*) responsible for L-proline biosynthesis and one gene (*TraesCS1D02G147800*) functioning in ABA-responsive signaling. RNA-seq-derived log_2_FoldChange values of these four genes are summarized in Figure 4d, and their shoot expression patterns quantified via qRT-PCR across three wheat cultivars are presented in Figure 4e–h. Overall, qRT-PCR expression trends were consistent with transcriptomic data, verifying the reliability of our DEG profiling. Relative to the CK group, all four genes displayed significantly altered transcript abundance under 150 mM salt stress. These qRT-PCR results thus corroborate the proposed roles of proline biosynthesis and ABA signaling in shoot osmotic adjustment under salt stress.

Altogether, the basal transcriptomic response of wheat seedlings at 150 mM NaCl suggests a classical “growth-defense trade-off” strategy: shoots prioritize the activation of osmotic adjustments, while roots exhibit heightened energy metabolism concurrent with significant cell cycle inhibition. Such a response pattern contributes to overall seedling growth retardation (as observed phenotypically in Section 2.1) and provides a crucial comparative framework for understanding specific tolerance strategies.

### 2.7. Identification of Jimai 60-Specific, Huapei 5-Specific, and Gaoyou 2018-Specific DEGs

Based on the classification method (Section 3.5), to explore the potential mechanisms underlying specific phenotypic divergence, we further analyzed the specific DEGs in the three selected wheat cultivars. Jimai 60 possessed 2254 (1275 up-regulated, 979 down-regulated) and 2670 (1160 up-regulated, 1510 down-regulated) specific DEGs in the shoots and roots, respectively (Figure S2a; Table S14). Huapei 5 possessed 4618 (2139 up-regulated, 2479 down-regulated) and 5510 (2412 up-regulated, 3098 down-regulated) specific DEGs in the shoots and roots, respectively (Figure S2b; Table S14). Gaoyou 2018 possessed 598 (170 up-regulated, 428 down-regulated) and 2152 (1168 up-regulated, 984 down-regulated) specific DEGs in the shoots and roots, respectively (Figure S2c; Table S14).

### 2.8. GO Enrichment Analysis of Jimai 60-Specific DEGs

**Figure 5:**
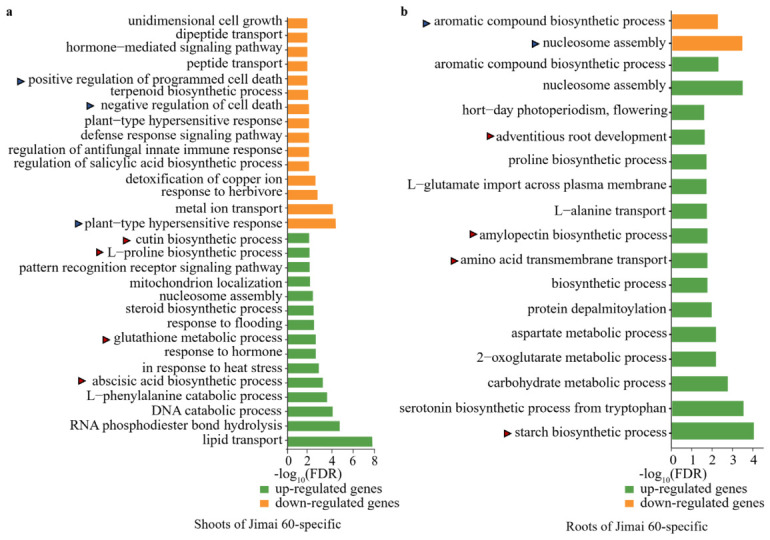
Specific differentially expressed genes (DEGs) of Jimai 60 under 150 mM salt treatment. (**a**,**b**) GO enrichment analysis of specific DEGs in shoots (**a**) and roots (**b**) of Jimai 60 under 150 mM salt treatment compared with the control (0 mM), identified by comparison with the other two cultivars (Huapei 5 and Gaoyou 2018). Green and yellow bars represent up-regulated and down-regulated DEGs, respectively. *p*-values were adjusted by the Benjamini–Hochberg correction, and only statistically significant GO categories (FDR < 0.05) are shown.

GO enrichment results showed that, in addition to ABA and L-proline biosynthesis, genes specifically up-regulated in Jimai 60 shoots were significantly enriched in cutin biosynthetic processes and glutathione metabolic processes (Figure 5a). This suggests that Jimai 60 shoots may reinforce physical shoot barriers by synthesizing cutin to reduce non-stomatal water loss, while simultaneously enhancing ROS scavenging capacity through glutathione metabolism to mitigate oxidative damage. Conversely, specifically down-regulated genes in Jimai 60 shoots were significantly enriched in the positive regulation of programmed cell death, plant-type hypersensitive response, and the negative regulation of unidimensional cell growth (Figure 5a). Contextualized with the stress-induced growth arrest observed across most accessions (Section 2.6), the specific down-regulation of cell death and growth-negative pathways in Jimai 60 may help inhibit apoptosis and partially maintain leaf elongation under adversity. These specific transcriptomic signatures provide a plausible molecular explanation for the salt tolerance of Jimai 60 shoots.

Unlike the shoots, the specific responses in Jimai 60 roots displayed an alternative transcriptional profile. Specifically, down-regulated genes were significantly enriched in nucleosome assembly and aromatic compound biosynthetic processes (Figure 5b). As noted in Section 2.6, activation of the nucleosome assembly is a basal response for DNA repair under salt stress; the specific down-regulation of this pathway in Jimai 60 likely weakens its basal capability to repair DNA damage. Concurrently, the suppression of aromatic compound (e.g., lignin precursors) synthesis suggests compromised structural barrier integrity. On the other hand, specifically up-regulated genes in Jimai 60 roots were heavily enriched in starch biosynthetic processes, amylopectin synthesis, and the transmembrane transport of certain amino acids (e.g., glutamate, alanine) (Figure 5b). This implies that stress induced a shift in carbon and nitrogen metabolism in Jimai 60 roots, leading to abnormal starch accumulation. Additionally, the specific up-regulation of “adventitious root development” may serve as a morphological compensation following primary root damage. The suppression of these basal defense-related pathways and the alteration in carbon metabolism turnover are likely primary reasons for the relatively lower salt tolerance of Jimai 60 roots. Together, these findings suggest that the divergent phenotype of Jimai 60 stems from different transcriptional response strategies: shoots primarily maintain physiological homeostasis by reinforcing physical/antioxidant barriers and suppressing apoptosis, whereas roots exhibit greater susceptibility due to compromised DNA assembly/repair pathways and altered carbon allocation.

### 2.9. GO Enrichment Analysis of Huapei 5-Specific DEGs

**Figure 6:**
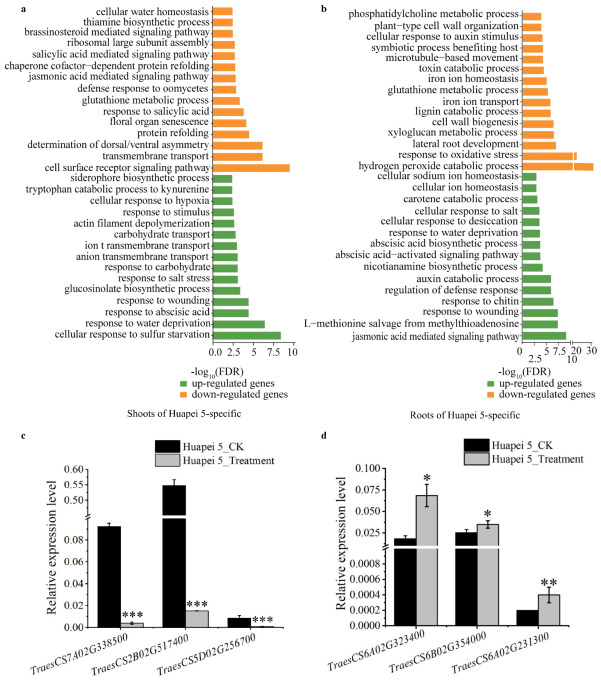
Specific differentially expressed genes (DEGs) of Huapei 5 under 150 mM salt treatment. (**a**,**b**) GO enrichment analysis of specific DEGs in shoots (**a**) and roots (**b**) of Huapei 5 under 150 mM salt treatment compared with the control (0 mM), identified by comparison with the other two cultivars (Jimai 60 and Gaoyou 2018). Green and yellow bars represent up-regulated and down-regulated DEGs, respectively. *p*-values were adjusted by the Benjamini–Hochberg correction, and only statistically significant GO categories (FDR < 0.05) are shown. (**c**) Expression patterns of three genes related to SA and JA signaling pathways determined by qRT-PCR in Huapei 5 shoots under salt stress. (**d**) Expression patterns of three genes related to cellular sodium ion homeostasis determined by qRT-PCR in Huapei 5 roots under salt stress. The expression of *ACTIN* was used to normalize mRNA levels. The values are means (±SE) of three biological replicates. *, ** and *** indicate significant differences at *p* < 0.05, *p* < 0.01 and *p* < 0.001, respectively, compared with the 0 mM control.

In the shoots, specifically up-regulated genes in Huapei 5 were significantly enriched in the regulation of ion transmembrane transport and responses to ABA and water deprivation (Figure 6a). This indicates that alongside basal osmotic adjustment, Huapei 5 shoots may actively promote the efflux or vacuolar compartmentalization of toxic ions, thereby alleviating ionic toxicity within the leaves. Concurrently, its specifically down-regulated genes were concentrated in salicylic acid (SA) and jasmonic acid (JA)-mediated signaling pathways, defense responses to oomycetes, and floral organ senescence (Figure 6a). We further performed qRT-PCR on enriched genes of SA and JA pathways, including two genes involved in both SA and JA signaling pathways (*TraesCS7A02G338500*, *TraesCS2B02G517400*) and one SA signaling-related gene (*TraesCS5D02G256700*), and the results verified their down-regulated expression, consistent with the RNA-seq data (Figure 6c). During abiotic stress, plants frequently activate pathogen immune responses due to signal crosstalk; the specific suppression of these immune and senescence pathways in Huapei 5 likely minimizes non-essential energy expenditures, preserving metabolic resources to sustain normal leaf physiological functions.

In the roots, which directly contact the saline environment, Huapei 5 exhibited distinct transcriptomic features related to ion homeostasis and morphological adaptation. Its specifically up-regulated genes were intensely enriched in cellular sodium ion homeostasis and general cellular ion homeostasis (Figure 6b). Several candidate genes (*TraesCS6A02G323400*, *TraesCS6B02G354000* and *TraesCS6A02G231300*) within the cellular sodium ion homeostasis pathway were further selected for qRT-PCR validation to confirm the transcriptomic results (Figure 6d). Maintaining sodium homeostasis is a critical rate-limiting step for salt tolerance. The specific activation of this pathway demonstrates that Huapei 5 roots possess superior sodium efflux or compartmentalization capabilities, mitigating Na^+^ toxicity at the source. Furthermore, its specifically down-regulated genes were involved in plant-type cell wall organization, responses to auxin, and the negative regulation of lateral root development (Figure 6b). This suggests that under salt stress, Huapei 5 may slow primary root elongation (suppressing cell wall and auxin responses) while concurrently releasing restrictions on lateral root development to modify its root architecture. This potential morphological adjustment could facilitate better adaptation to high-salinity substrates.

Overall, the “dual-tolerant” phenotype of Huapei 5 is likely driven by a multi-dimensional regulatory network: its roots focus on activating sodium homeostasis and adaptive root architecture modifications, while its shoots maintain physiological balance by promoting ion transmembrane transport and strategically suppressing non-essential immune responses. These results provide an important theoretical reference for understanding the synergistic mechanisms of shoot and root salt tolerance in wheat.

### 2.10. GO Enrichment Analysis of Gaoyou 2018-Specific DEGs

In shoots, the specifically up-regulated genes in Gaoyou 2018 were significantly enriched only in the glutamate catabolic process, with low enrichment significance (Figure S3a). This pathway is mainly associated with basic energy supply rather than specific salt tolerance regulatory mechanisms. Meanwhile, the specifically down-regulated genes were significantly enriched in pathways related to stress signal transduction and structural defense, including protein phosphorylation, stress-activated kinase signaling cascades, the brassinosteroid-mediated signaling pathway, and plant-type secondary cell wall biogenesis. These results indicate that core salt stress signaling is deficient, and both hormone-mediated defense pathways and the cell wall structural barrier system may be disrupted in shoots of Gaoyou 2018 (Figure S3a).

In roots, the specifically up-regulated genes in Gaoyou 2018 were significantly enriched in stress response, such as the response to water deprivation, glutathione metabolism, lignin biosynthesis, and anion transmembrane transport, but the enrichment degree was relatively low. In addition, the specifically down-regulated genes in roots were enriched in pathways associated with nutrient transport and cellular homeostasis (Figure S3b). These results indicate that although the root attempted to initiate stress defense responses preferentially, the corresponding enrichment level remained weak, while root growth and nutrient uptake processes were still inhibited.

Overall, the dual-sensitive phenotype of Gaoyou 2018 may be attributed to disordered and inefficient regulatory networks: its roots initiate weak stress defense responses with limited enrichment, while its shoots suffer from deficient salt stress signaling and disrupted structural defense systems.

## 3. Discussion

### 3.1. Importance of Selecting the Right Concentration to Identify Extreme Germplasm

Salt tolerance in wheat is a complex trait controlled by many genes. Previous studies have noted that due to differences in testing concentrations and evaluation methods [20,21], population screening often cannot fully reveal the true genetic variation in tolerance among different wheat accessions. Our study confirmed that using 150 mM as the appropriate salt stress concentration effectively reduces false positives and clearly shows the phenotypic variations within the population, which agrees with findings in other plant species [22,23]. More importantly, our data showed continuous distribution in the length of shoots and roots’ response to salt stress at the population level. Using a two-dimensional evaluation model based on “shoot × root” resistance, we found that > 40% of the accessions in the natural population showed obvious “organ-specific differences”. This suggests that during the seedling stage, wheat’s response to salt stress is tissue-specific. Relying only on a single whole-plant measurement, such as total biomass or survival rate, can easily hide important genetic resources [20]. Analysis of overlapping and organ-specific DEGs revealed obvious transcriptional divergence between shoot and root, which further supported a typical growth–defense trade-off strategy upon salt stress.

### 3.2. A Possible “Growth-Defense Trade-Off” Mechanism in Wheat Responding to Salt Stress

The striking discrepancy in overlapping DEGs between shoots and roots could be largely explained by the conserved plant growth–defense trade-off strategy under salt stress. Removing background noise to find the shared regulatory basis (overlapping DEGs) is a key step for understanding the molecular mechanisms of salt tolerance [24]. Based on the shared gene expression data, our study observed a pattern consistent with “growth-defense trade-off” in plants dealing with salt stress [25,26]. On the one hand, after salt exposure, the shoots of all tested wheat accessions activated dehydration responses controlled by ABA and osmotic adjustment pathways mainly driven by proline accumulation [17,18,19,27]. At the same time, in the roots—the organ directly contacting the salt—genes responsible for cell division, DNA replication, and chromatin assembly, which directly promote growth, were consistently down-regulated [28]. This result is in line with the classical “growth arrest” under abiotic stress: under severe environmental stress, plants tend to restrict high-energy growth processes to save more resources for basic survival and defense [29,30,31]. The presence of this “basal defense” likely explains the unavoidable whole-plant growth reduction observed in all accessions under the 150 mM stress treatment. We acknowledge that this interpretation is based primarily on transcriptomic data and requires further experimental validation.

### 3.3. Excellent Performance of Jimai 60 in Shoots Under Salt Stress

By analyzing the specific gene expression of Jimai 60, we found that its phenotype—showing higher tolerance in shoots than in roots—is controlled by different regulatory strategies. In the shoots, this accession specifically up-regulated the synthesis of cutin- and glutathione-related antioxidant responses, while down-regulating pathways that cause cell death and limit cell growth. These findings suggest that its higher tolerance in the shoots comes from stronger physical barriers and effective ROS clearing [32,33]. However, this stable state in the shoots cannot make up for the severe damage in the root system. Jimai 60 roots showed a decrease in aromatic compound (lignin) synthesis and weakened DNA assembly processes. The suppression of these basic defense pathways not only stops water and nutrient absorption due to root damage but will eventually break the shoot–root balance, making its overall salt tolerance hard to maintain over time.

### 3.4. The Core Regulatory Network of the Dual-Tolerant Accession Huapei 5

The dual-tolerant accession Huapei 5 maintained high activity in both shoots and roots, showing a more complete salt resistance network. The main molecular feature of this accession was the strong activation of “sodium ion homeostasis” pathways in its roots. Pumping Na^+^ out of the cell or storing it in vacuoles (for example, using SOS1 or NHX transporters) is a key step to keep cell toxicity low and ensure plant survival [34,35]. The efficient activation of this ion balance network in Huapei 5 roots successfully blocked the transport of salt to the shoots. Additionally, its suppression of genes that limit lateral root development suggests that this accession might improve its adaptability by changing its root shape [36]. Meanwhile, in the shoots, besides activating basic osmotic adjustments, Huapei 5 specifically down-regulated SA and JA pathways, as well as general immune responses against pathogens. Under severe salt stress, actively reducing unnecessary disease resistance responses is a well-known “energy-saving strategy” [37,38]. By saving the energy normally used for these secondary defenses and using it to maintain core survival functions, this energy reallocation system is likely the key reason why Huapei 5 grows so well in highly saline environments.

### 3.5. Differential Regulation of Salt-Responsive Pathways in Three Wheat Cultivars

Maintaining Na^+^ homeostasis and modulation of reactive oxygen species (ROS) production and signal specificity have been reported to be important salt tolerance mechanisms in wheat [39]. Furthermore, ABA is well established as a central phytohormone governing plant salt stress responses, orchestrating osmotic adjustment, ion homeostasis, and ROS scavenging [40]. These reports are highly consistent with the results observed in our GO enrichment analysis of overlapping DEGs under salt stress in different tissues of the three cultivars Huapei 5, Jimai 60, and Gaoyou 2018, particularly regarding the regulation of ABA and response to salt stress. In addition, we further compared and analyzed GO-enriched pathways derived from specific DEGs in three cultivars. The Huapei 5 exhibited significant enrichment in various abscisic acid pathways, together with up-regulated genes involved in cellular response to salt and cellular Na^+^ homeostasis pathways, which contributed to its excellent salt tolerance. Jimai 60 showed significant enrichment in the abscisic acid biosynthetic process along with ROS regulation (glutathione metabolic processes) [41], which may be associated with its divergent phenotype. Whereas Gaoyou 2018 lacked effective core pathways for salt stress response in shoots, only the glutathione catabolic process (ROS regulation) was enriched in roots, which likely explains its dual-sensitive phenotype.

This transcriptome analysis revealed genotypic differences in salt stress responses among wheat accessions and provided valuable insights into tissue-specific and genotype-dependent salt-responsive pathways in wheat. It should be noted that the present study is primarily based on transcriptomic analysis, and quantitative measurements of physiological indicators under salt stress were not performed. In future research, we will prioritize the determination of phytohormone contents (ABA, SA, and JA), ion homeostasis (including Na^+^ and K^+^ content), and ROS levels, combined with the use of corresponding near-isogenic lines, to conduct in-depth experiments on the candidate pathways and key genes identified in this study, thereby further elucidating the molecular mechanisms underlying salt tolerance in wheat.

## 4. Materials and Methods

### 4.1. Plant Materials and Growth Conditions

Six wheat cultivars (Zhoumai 27, Jinan 17, Jimai 60, Jimai 22, Aifeng 3, and Lumai 23) were used to screen for appropriate salt stress concentrations, and 137 natural wheat accessions were subjected to salt tolerance analysis at the seedling stage. Seeds were surface-sterilized with 2% sodium hypochlorite for 30 min, placed in Petri dishes lined with filter paper, and moistened with distilled water. The seeds were incubated at 4 °C in darkness for 3 d to induce germination [42]. Seedlings were then transplanted into hydroponic boxes (127 mm × 127 mm × 114 mm) and grown in a constant-temperature incubator at 22 °C for another 1 d to synchronize germination. Distilled water was added regularly to maintain adequate moisture.

Uniformly germinated seeds were selected and transferred to hydroponic culture systems. The hydroponic culture was performed using Hoagland nutrient solution, prepared with commercial calcium-free Hoagland nutrient powder (Cat No. HB8870-1, Hopebio, Qingdao, China) following the manufacturer’s instructions. For the preparation of 1 L of Hoagland solution, 1.26 g of the calcium-free powder and 0.945 g of calcium nitrate tetrahydrate (Ca(NO_3_)_2_·4H_2_O) were weighed separately and then dissolved in 1 L of distilled water with gentle heating and stirring to avoid precipitation. After complete dissolution, the solution was sterilized by autoclaving (115 °C, 20 min) and stored in sterile bottles.

Plants were cultured in a constant-temperature incubator under a 16 h light/8 h dark photoperiod (light intensity > 12,000 lx, temperature of 22 °C) [43]. To ensure consistent salt stress intensity, nutrient supply, and pH stability, the nutrient solution was replaced every 3 d to minimize ion imbalance.

### 4.2. Screening of Optimal Salt Concentration

The Hoagland nutrient solution was used as the culture medium, and NaCl was added to the solution to prepare five salt concentration treatments with final concentrations of 0, 50, 100, 150, and 200 mM [44]. The specific calculation method for each salt concentration was as follows: based on the molar mass of NaCl (58.44 g/mol), the mass of NaCl required to prepare 1 L of nutrient solution with different concentrations was calculated (e.g., 2.922 g of NaCl was added to 1 L of nutrient solution to obtain a 50 mM NaCl concentration; 5.844 g for 100 mM, 8.766 g for 150 mM, and 11.688 g for 200 mM). All hydroponic boxes were then placed in a constant-temperature incubator. After 7 d of treatment, the wheat seedlings were harvested. Shoot length and maximum root length were measured using a ruler. Shoots and roots were then separated, and shoot fresh weight and root fresh weight were determined using an electronic balance. The root/shoot length ratio and root/shoot fresh weight ratio were calculated accordingly. All traits were measured for each treatment, with five biological replicates per treatment. Based on the coefficients of variation (CVs) of the salt tolerance index (STI) calculated from shoots and roots lengths, 150 mM NaCl was selected as the optimal concentration for subsequent experiments.

### 4.3. Phenotyping of Natural Accessions

Uniformly germinated seeds were transplanted into hydroponic boxes containing Hoagland nutrient solution supplemented with 0 mM NaCl (control) or 150 mM NaCl and grown in a constant-temperature incubator. After 7 d, shoots and roots were measured under both salt stress and control conditions, with five biological replicates per treatment. Given that this study focused on the differential salt tolerance responses between shoots and roots and aimed to minimize experimental random error, Jimai 60 was used as a common control variety across different batches of salt stress and control treatments due to its stable phenotype and distinct contrasting salt responses between the two parts. The STI of each accession was normalized to that of Jimai 60 to ensure comparability among different experimental batches.

### 4.4. Statistical Analysis

The STI [45] for each accession was calculated as follows:

$$\mathrm{STI}=\frac{\mathrm{Mean}\;\mathrm{value}\;\mathrm{under}\;\mathrm{salt}\;\mathrm{stress}}{\mathrm{Mean}\;\mathrm{value}\;\mathrm{under}\;\mathrm{control}\;\mathrm{condition}}$$

Around 30 samples were phenotyped per experimental batch. To ensure data comparability across experimental batches, the relative salt tolerance index (RSTI) was further calculated with Jimai 60 as the reference variety:

$$\mathrm{RSTI}=\frac{\mathrm{STI}\;\mathrm{of}\;\mathrm{tested}\;\mathrm{accession}}{\mathrm{STI}\;\mathrm{of}\;\mathrm{Jimai}\;60}$$

Descriptive statistical parameters, including the mean, standard deviation (SD), and CVs, were calculated using Microsoft Excel 2019.

### 4.5. Transcriptome Sequencing and Data Analysis

Transcriptome profiling was performed on the shoots and roots tissues of 7-day-old hydroponic seedlings of Huapei 5, Jimai 60, and Gaoyou 2018 under 0 mM and 150 mM NaCl treatments. Tissues were collected into separate centrifuge tubes, immediately frozen in liquid nitrogen, and stored at −80 °C. Three biological replicates were performed for each sample.

Total RNA was extracted using the Quick RNA Isolation Kit DP441 (Tiangen Biotech Co., Ltd., Beijing, China). Strand-specific RNA libraries were constructed via poly(A) mRNA enrichment, and RNA sequencing was conducted on the Illumina platform with 150 bp paired-end reads by a commercial service provider (Novogene Bioinformatics Technology Co., Ltd., Beijing, China). Clean reads were aligned to the IWGSC RefSeq v1.1 reference genome. To reduce low-expression noise, genes with an average fragments per kilobase per million fragments (FPKM) < 1 across all samples were excluded from downstream functional enrichment and visualization analyses. For differential expression analysis, raw read counts were used as input for DESeq2 (v1.24.0) [46], with low-count genes filtered using the independent filtering algorithm implemented in DESeq2 by default. Only the genes with an absolute log_2_(fold change) ≥ 1 and a false discovery rate (FDR) < 0.05 were considered significantly DEGs. Venn diagram analysis was performed using the online tool Venny 2.1.0 (Oliveros, J.C., BioinfoGP, CNB-CSIC; https://bioinfogp.cnb.csic.es/tools/venny/index.html, accessed on 23 March 2026). Significantly enriched gene ontology (GO) terms for up-regulated and down-regulated DEGs (FDR < 0.05) were identified using Triticeae-Gene Tribe (http://wheat.cau.edu.cn/TGT/, accessed on 23 March 2026) [47].

### 4.6. RT-qPCR

To verify the reliability of the transcriptome data, RT-qPCR validation was performed using identical tissue samples obtained from the same salt treatment and experimental batches described above for transcriptome sequencing. Total RNA was extracted from seedling shoots and roots of Huapei 5, Jimai 60, and Gaoyou 2018 under 0 mM and 150 mM NaCl conditions using the Quick RNA Isolation Kit DP441 (Tiangen Biotech Co., Ltd., Beijing, China). RNA integrity and purity were assessed before reverse transcription. First-strand cDNA was synthesized from 1 μg of total RNA using HiScript II Q RT SuperMix (Vazyme Biotech, Nanjing, China, R223-01) according to the manufacturer’s protocols.

The RT-qPCR reaction system was prepared using Taq Pro Universal SYBR qPCR Master Mix (Vazyme Biotech, Q712-02/03) in a total volume of 10 μL, containing 5 μL of SYBR Master Mix, 1 μL of diluted cDNA template, and 0.2 μM of each primer. The amplification procedure was conducted on a real-time PCR detection system (LightCycler 480, Roche Diagnostics, Rotkreuz, Switzerland). The wheat *ACTIN* gene (*TraesCS5B02G124100*) was used as the internal reference gene for normalization of gene expression levels. Relative expression values were calculated using the Ct (2^−ΔCT^) method. RT-qPCR was performed as technical triplicates per sample. Three biological replicates were performed, with similar results; the results from one replicate are shown in the figures. Significant differences are indicated by * *p* < 0.05, ** *p* < 0.01 and *** *p* < 0.001, as determined by Student’s *t* test. Primers are listed in Table S15.

## 5. Conclusions

In this study, we established an integrated shoot-and-root screening system at the seedling stage under an appropriate 150 mM salt stress concentration, which revealed organ-specific divergence in salt tolerance across a natural population of 137 wheat accessions. By integrating transcriptomic analysis of seedling tissues, we observed a physiological trade-off in wheat seedlings responding to salt stress, characterized by the prioritization of basal defense. The transcriptional networks of the representative phenotypes further demonstrated that the divergent accession Jimai 60 relies on reinforcing shoot barriers to maintain local stay-green traits, whereas the suppression of root metabolism and defense limits its overall tolerance. Conversely, the dual-tolerant accession Huapei 5 exhibits systemic adaptation; it up-regulates networks associated with sodium ion homeostasis in roots while attenuating non-essential immune responses at the shoot and root level to facilitate defense energy reallocation. As a typical dual-sensitive accession, Gaoyou 2018 may have failed to effectively activate key salt tolerance-related pathways in both shoots and roots, instead showing widespread suppression of core defense pathways. The contrasting germplasm and specific transcriptional regulatory modules identified herein offer new insights for understanding salt tolerance networks in wheat at the seedling stage. In future research, we will prioritize physiological measurements together with near-isogenic lines to validate the candidate pathways and key genes identified in this study.

## Data Availability

RNA sequencing data are available at the National Center for Biotechnology Information Sequence Read Archive (SRA) under BioProject accession number PRJNA1446960 (https://www.ncbi.nlm.nih.gov/bioproject/PRJNA1446960, accessed on 2 June 2026).

## Supplementary Materials

The following supporting information can be downloaded at: https://www.mdpi.com/article/10.3390/plants15121905/s1, Figure S1. Statistics of differentially expressed genes (DEGs) in three representative wheat cultivars under 150 mM salt treatment. Figure S2. Numbers of specific differentially expressed genes (DEGs) in wheat cultivars under 150 mM salt treatment. Figure S3. Specific differentially expressed genes (DEGs) of Gaoyou 2018 under 150 mM salt treatment. Table S1. Statistical analysis of phenotypic traits for six cultivars under five salt stress levels. Table S2. Statistical analysis of salt tolerance index in six cultivars under four salt stress levels. Table S3. Statistical analysis of the overall variation range of 137 accessions under 150 mmol/L salt stress. Table S4. Statistical analysis of phenotypic traits of 137 accessions under 150 mmol/L salt stress. Table S5. Summary of transcriptome data. Table S6. Statistical summary of DEGs for three representative cultivars. Table S7. Differentially expressed genes in shoots of Gaoyou 2018 under 150 mM salt treatment vs. CK (0 mM). Table S8. Differentially expressed genes in roots of Gaoyou 2018 under 150 mM salt treatment vs. CK (0 mM). Table S9. Differentially expressed genes in shoots of Huapei 5 under 150 mM salt treatment vs. CK (0 mM). Table S10. Differentially expressed genes in roots of Huapei 5 under 150 mM salt treatment vs. CK (0 mM). Table S11. Differentially expressed genes in shoots of Jimai 60 under 150 mM salt treatment vs. CK (0 mM). Table S12. Differentially expressed genes in roots of Jimai 60 under 150 mM salt treatment vs. CK (0 mM). Table S13. Overlap DEGs from shoots and roots tissues, respectively, among Huapei 5, Jimai 60, and Gaoyou 2018 under 150 mM salt treatment vs. control (0 mM). Table S14. Genotype-specific DEGs in shoots and roots tissues among Huapei 5, Jimai 60, and Gaoyou 2018 under 150 mM salt treatment vs. control (0 mM). Table S15. Primers used for gene expression analysis.

## Abbreviations

The following abbreviations are used in this manuscript:

DEGs	Differentially expressed genes
ROS	Reactive oxygen species
CVs	Coefficients of variation
STI	Salt tolerance index
RSTI	Relative salt tolerance index
SD	Standard deviation
FPKM	Fragments per kilobase per million fragments
FDR	False discovery rate
GO	Gene ontology
PCA	Principal component analysis
ABA	Abscisic acid
SA	Salicylic acid
JA	Jasmonic acid
