# Supplementary material for: Phenotypic Screening and Organ-Specific Transcriptomics Unveil Diverse Salt Tolerance Responses at the Seedling Stage in Wheat (Triticum aestivum L.)
Source: Plants (Basel). 2026 Jun 19;15(12):1905. doi: 10.3390/plants15121905 (PMC13306350; doi:10.3390/plants15121905)
Supplement: Supplementary file 1 [file plants-15-01905-s001.zip › Supplementary Figures-0615.pdf]

Supplementary Figures

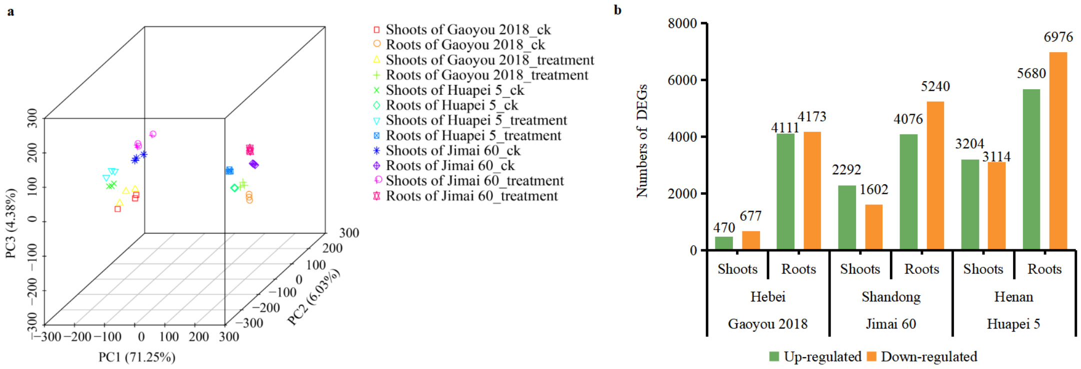

Figure S1. Statistics of differentially expressed genes (DEGs) in three representative wheat cultivars under 150 mM salt treatment compared with control (0 mM). (a) Principal component analysis (PCA) of transcriptome data from shoots and roots of the three cultivars under 150 mM salt treatment compared with the control (0 mM); (b) Number of up-regulated and down-regulated DEGs in shoots and roots of the three cultivars under 150 mM salt treatment compared with the control (0 mM). Green and yellow bars represent up-regulated and down-regulated DEGs, respectively.

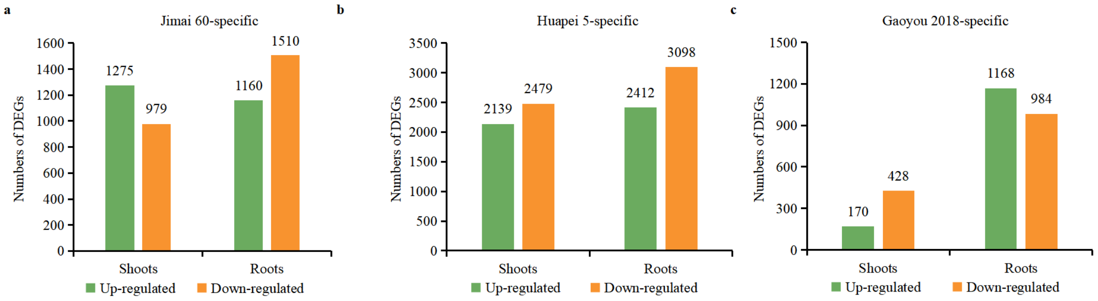

Figure S2. Number of specific differentially expressed genes (DEGs) in wheat cultivars under 150 mM salt treatment compared with control (0 mM). (a, b) Number of specific DEGs in shoots and roots of Jimai 60 (a), Huapei 5 (b) and Gaoyou2018 (c) under 150 mM salt treatment compared with the control (0 mM), identified by comparison with the other two cultivars. Green and yellow bars represent up-regulated and down-regulated DEGs, respectively.

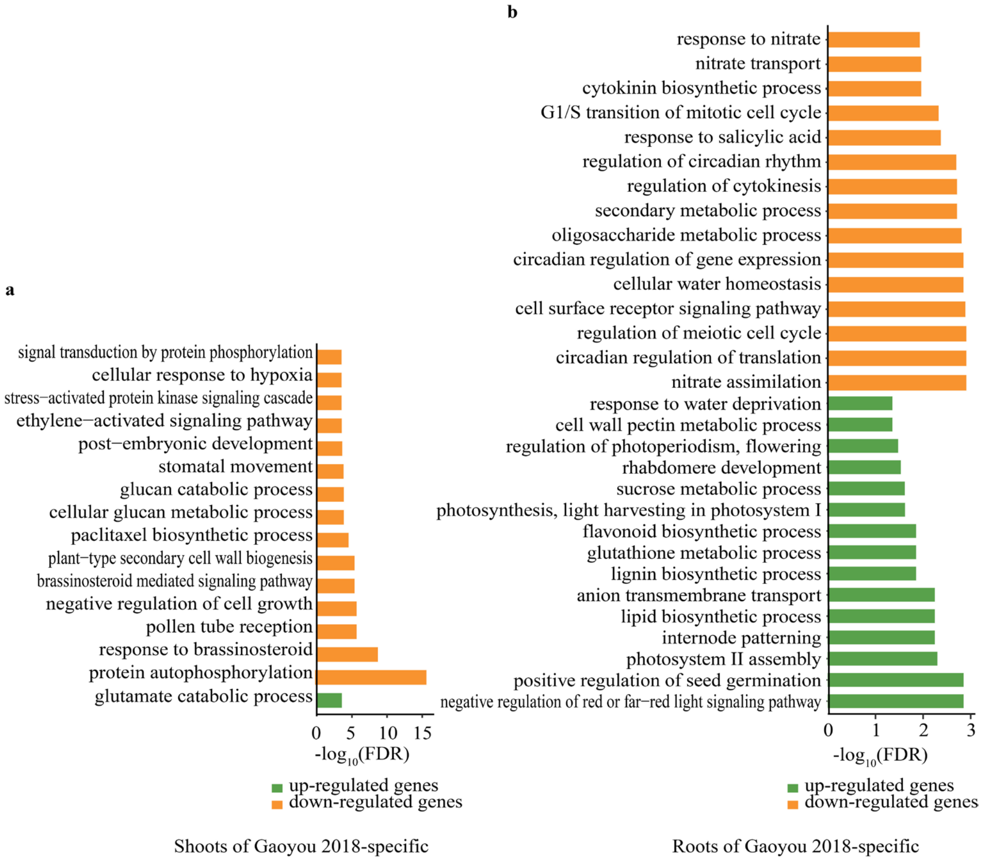

Figure S3. Specific differentially expressed genes (DEGs) of Gaoyou 2018 under 150 mM salt treatment. (a, b) GO enrichment analysis of specific DEGs in shoots (a) and roots (b) of Gaoyou 2018 under 150 mM salt treatment compared with the control (0 mM), identified by comparison with the other two cultivars (Jimai 60 and Huapei 5). Green and yellow bars represent up-regulated and down-regulated DEGs, respectively. *P*-values were adjusted by the Benjamini-Hochberg correction, and only statistically significant GO categories (FDR < 0.05) are shown.
